# Acceptability of HPV vaccination for cervical cancer prevention amongst emerging adult women in rural Mysore, India: a mixed-methods study

**DOI:** 10.1186/s12889-024-19485-8

**Published:** 2024-08-07

**Authors:** Kate Coursey, Kiranmayee Muralidhar, Vijaya Srinivas, Poornima Jaykrishna, Fazila Begum, Nagalambika Ningaiah, Sung-Jae Lee, Purnima Madhivanan

**Affiliations:** 1 Department of Medicine, David Geffen School of Medicine at UCLA, Los Angeles, CA 90095 USA; 2 Public Health Research Institute of India, Mysore, Karnataka 570020 India; 3 JSS Academy of Higher Education and Research, Mysuru, Karnataka 570004 India; 4 Department of Psychiatry and Biobehavioral Sciences, David Geffen School of Medicine at UCLA, Los Angeles, CA 90095 USA; 5 Department of Health Promotion Sciences, Zuckerman College of Public Health, University of Arizona, Mel & Enid, Tucson, AZ 85724 USA

**Keywords:** HPV, Cervical cancer, India, Vaccination, Vaccine acceptability, Conjoint analysis

## Abstract

**Background:**

India has the highest number of estimated deaths from cervical cancer globally, with most cases attributed to Human papillomavirus (HPV). The World Health Organization recommends primary HPV vaccination for girls ages 9–14, with catch-up vaccination for young women ≥ 15 if feasible. India authorized a new, inexpensive HPV vaccine in 2022; given anticipated vaccine expansion, we conducted a mixed-methods study exploring acceptability of HPV catch-up vaccination for young emerging adult women in rural Mysore, India.

**Methods:**

Between September 2022-April 2023, participants were recruited with assistance from community health workers. In the qualitative phase, gender-stratified, audio-recorded focus group discussions (FGDs) were conducted in *Kannada* with emerging adults ages 18–26. FGDs were transcribed, translated, and analyzed using rapid approach to identify key HPV vaccination attributes. In the quantitative phase, a conjoint analysis was conducted to assess the impact of seven vaccination attributes on likelihood to vaccinate (LTV). Women ages 18–26 ranked LTV in eight hypothetical vaccination scenarios, and the relative impact of each attribute on LTV was calculated. All participants received education about cervical cancer, HPV, and HPV vaccination.

**Results:**

Fifty-two young adults (female = 31, male = 21) participated in seven FGDs, and 101 women participated in the conjoint analysis. Average age of the 153 participants was 22.5 years, 66.7% had married, and all had completed high school. Only 17.9% had heard of cervical cancer, and 2.7% knew of the HPV vaccine. FGDs identified seven HPV vaccination attributes: cost, vaccination location, family support, peer influence, dose number, side effects, and risk of acquiring HPV. In the conjoint analysis, all attributes except dose number significantly impacted LTV. Family support (impact score = 19.37, *p* < 0.0001) and peer influence (impact score = 18.01, *p* < 0.0001) had the greatest influence, followed by cost (impact score = 16.64, *p* < 0.0001) and HPV risk (impact score = 12.31, *p* < 0.0001). Vaccination location (government centers preferred) and side effects were also significant.

**Conclusion:**

Participants had poor knowledge of cervical cancer and HPV. Social attributes (family support, peer influence) had greatest impacts on LTV, and future studies should explore family-based interventions and peer education. Providing free vaccines at government centers through India’s national immunization program would maximize catch-up HPV vaccination for rural young women.

## Introduction

Cervical cancer is the second most common cancer affecting women in India, with nearly 128,000 new cases and 80,000 deaths yearly [1, 2]. Most cervical cancers arise from persistent infection with Human Papillomavirus (HPV), a common sexually transmitted infection (STI) [3]. While HPV vaccines have been approved for Indian school-aged girls since 2008, the government of India temporarily halted vaccine studies in 2010 after the deaths of seven young girls who received HPV vaccination through implementation projects in the states of Gujarat and Andhra Pradesh [4]. Although further investigation revealed these deaths were unrelated to the HPV vaccine, misconceptions about side effects have continued to impede support for vaccination campaigns over the past decade [5, 6]. Additionally, high cost of the HPV vaccine has been a major barrier to uptake in India and other low- and middle-income countries [7–9].

Two recent developments promise to reduce the financial burden of HPV vaccination in India. First, the Serum Institute of India, a prominent vaccine manufacturer in India, launched a new, indigenously-developed HPV vaccine in September 2022 at significantly cheaper cost per-dose than existing vaccines; the government subsequently announced a gradual roll-out for adolescent girls as part of India’s National Immunization Program [10]. Second, a 2022 update from the World Health Organization (WHO) suggested an HPV vaccination schedule with reduced number of doses can effectively prevent infection [11]. WHO guidelines recommend vaccination for girls ages 9–14 as a primary target population. Young women over age 15 are a secondary target population for catch-up vaccination if affordable and feasible, [11] and in many countries, the upper age cutoff for routine vaccination is 26 [12–15]. India’s initial roll-out of the HPV vaccine will focus on the primary target population of girls 9–14 years; [10] however, as cheaper HPV vaccines enter the market with reduced-dose schedules, vaccinating older adolescents and young women will become more financially viable in India. Catch-up vaccination in this secondary target population has been shown to decrease HPV infection rates, [16, 17] and economic modeling suggests that inclusion of catch-up vaccination for older women can be a cost-effective way to decrease cervical cancer burden [18, 19].

With anticipated expansion of HPV vaccination, it is critical to understand potential uptake in women over 15 years in India. Few studies have specifically examined vaccine acceptability in adult women ages 18–26, who are vaccine-eligible but too old to be reached through traditional, school-based HPV vaccine campaigns [20]. In a 2016 survey of Indian college students, only 46% of women knew that HPV was related to cervical cancer, while 44% knew the vaccine existed [21]. Knowledge is likely even poorer amongst less educated women and women from rural areas [22–25]. Cultural factors, including stigma surrounding STIs and fear of judgment from family and community members, may negatively impact vaccine acceptability [7, 24]. Other barriers include cost, fear of side effects, perceived lack of need, and questions of vaccine efficacy [24, 26]. Among married women, spousal support or lack thereof additionally impacts vaccination and healthcare utilization [27, 28].

We used mixed methods to explore HPV vaccine acceptability amongst young women living in rural villages in Mysore District, India. Our first aim was to qualitatively assess knowledge of HPV and its association with cervical cancer, knowledge of the HPV vaccine, and barriers/facilitators to HPV vaccination in this population; our second aim was to quantitatively measure the impact of key vaccination attributes on women’s decision to vaccinate. These results may inform targeted, resource-conscious interventions to reach women in rural areas for catch-up HPV vaccination.

## Methods

### Overview

Qualitative data for our mixed methods study were collected through gender-stratified focus group discussions (FGDs) with young women and young men. Young men were included in the qualitative phase because their opinions as husbands, brothers, and fathers may impact women’s vaccine uptake.

Quantitative data were collected through conjoint analysis, a marketing analysis technique that evaluates the relative impact of various HPV vaccination attributes (i.e. cost, number of doses, side effects) on women’s likelihood to vaccinate (LTV). While conjoint analysis has been previously applied to vaccine and other biomedical research, [29–32] it has not been used to assess HPV vaccine acceptability in India. In conjoint analysis, participants are presented with eight hypothetical vaccination scenarios, each consisting of different combinations of desirable and non-desirable attributes. Participants rate LTV for each scenario, which allows for quantification of the relative impact of each attribute on vaccination intention.

#### Study Site and Population

Participants for this mixed-methods study were drawn from rural villages in Mysore District in the state of Karnataka, India, where *Kannada* is the official regional language. As of the most recent census, Mysore District had a population of just over 3 million people, of which almost 1.5 million were female; 58.5% lived in rural villages and 95.6% of the rural population identified as Hindu [33]. In the entire state, 20.5% of women aged 15–19 have ever married, increasing to 66.8% for women aged 20–24 and 89.8% for women aged 25–29 [33]. The most recent National Family Health Survey found a literacy rate of 73% for women in Karnataka [34].

#### Ethics Statement

This study was conducted by the Public Health Research Institute of India (PHRII), a nonprofit organization that has been conducting community-based research and providing health services in Mysore for the past 15 years. This study was approved by PHRII’s Institutional Ethics Review Board and by the University of California Los Angeles Institutional Review Board. Only women who were able to give informed consent were recruited to participate in the study.

#### Participant Recruitment

Between September 2022-November 2022, participants were recruited from rural villages in Mysore District for focus group discussions. Villages were identified from the pool of rural communities where PHRII has previously worked, then the PHRII outreach team contacted community health workers from each village to assist with identifying and recruiting eligible participants. Participants met with PHRII staff in the community to learn more about the study and to confirm eligibility. For those interested in participating, a follow-up meeting was arranged to conduct a FGD. Conjoint analysis participants were recruited from March 2023-April 2023 in a similar process to FGD participants via convenience sampling with assistance from local community health workers. After participants underwent eligibility screening, a follow-up meeting was arranged to conduct the conjoint analysis.

Inclusion criteria for FGD were: 1) men and women aged 18–26; 2) able to speak *Kannada*; 3) willing to be audio recorded; and 4) have the ability to undergo informed consent process. Inclusion criteria for conjoint analysis were: 1) women aged 18–26; 2) able to speak *Kannada* or English; 3) never received the HPV vaccine; and 4) have the ability to undergo informed consent process. A lower age limit of 18 was chosen because we wished to focus on young women who would not be reached through school-based vaccination campaigns; an upper age limit of 26 was chosen because HPV catch-up vaccination is often given up to this age. Pregnant women were excluded because the HPV vaccine is not currently recommended during pregnancy.

#### Data Collection and Analysis – FGDs

Gender-stratified FGDs took place in community spaces where privacy was ensured or at the PHRII office. FGD participants underwent informed consent process, and answered a short survey that collected basic demographic information and prior knowledge of cervical cancer and HPV. Audio-recorded FGDs were conducted using a semi-structured interview guide by a trained qualitative research assistant in *Kannada*. Guide questions were developed using the Increasing Vaccination Model (IVM) as a theoretical framework, which postulates that what people think and feel, social processes, and direct behavioral changes are the three primary factors driving vaccination [35, 36]. This model was recently adapted by the WHO as the WHO behavioural and social drivers of vaccination framework (BeSD framework) [37]. We chose IVM because it highlights the structural and social factors contributing to vaccination intention, in addition to individual-level attitudes and beliefs. Sample FGD questions included the following: “In your opinion, what are the qualities of a good vaccine?” and “What are some reasons that might make you hesitate/would make you more likely to receive the HPV vaccine?”.

FGDs consisted of two parts. During the first part, participants discussed general beliefs and attitudes about vaccination, and any prior knowledge of cervical cancer and HPV; afterward, the audio recording was paused and the research assistant provided 20 minutes of education on cervical cancer, HPV, and HPV vaccination using an illustrated flip book and a factsheet adapted and translated from WHO materials [11, 38, 39]. After providing education, the audio recording was restarted for the second part of the discussion, which focused on potential barriers and facilitators to HPV vaccination for young women.

Audio recordings were transcribed and translated to English, and rapid qualitative analysis was used to analyze FGD data [40]. Based on a review of discussion guide questions and translated transcripts, key domains were identified for women’s and men’s FGDs. These domains were incorporated into a summary template, and two authors (KC and KM) independently summarized each FGD using bullet point format. Summaries were discussed amongst the authors to reach consensus, then bullet points were transferred into matrices to examine findings for each domain across all FGDs. Recurring “attributes” related to HPV vaccination were identified from thematic analysis of each domain in the FGD matrices.

#### Data Collection and Analysis – Conjoint Analysis

Based on analysis of FGD data, seven HPV vaccination attributes were selected for further exploration through conjoint analysis, and two dichotomous levels (one preferred, one non-preferred) were assigned to each attribute as follows: cost (free vs. INR 1,000 rupees (approximately 12 U.S. dollars (USD)), location (government hospitals and subcenters vs. private clinics and hospitals), family support (none vs. support), peer influence (knows peers who are vaccinated vs. does not know any vaccinated peers), dose number (one vs. two), side effects (none vs. minor (fever, nausea, joint pain)), and risk of getting HPV (high risk vs. low risk). While seven dichotomous attributes can produce up to 128 unique attribute combinations, conjoint analyses commonly utilize an eight-run Plackett–Burman fractional factorial design to reduce this number to eight (Table 1) [41]. Each of the eight vaccination scenarios (conjoints) generated from this design contained a different combination of attributes, some preferred and some non-preferred. This design allowed us to estimate the main effects of each attribute, assuming that there were no interactions between the attributes [42].

**Table 1:** Conjoint analysis experimental design for HPV vaccination among young adult women in rural Mysore, India

	*Attributes*						
**HPV Vaccination Scenarios**	**Cost**	**Location**	**Family support**	**Peer influence**	**Dose number**	**Side effects**	**HPV risk**
1	1000 INR	Government hospitals	None	No vaccinated peers	1	None	High
2	Free	Government hospitals	None	Vaccinated peers	2	None	Low
3	1000 INR	Private hospitals	None	Vaccinated peers	1	Yes—Mild	Low
4	Free	Private hospitals	None	No vaccinated peers	2	Yes—Mild	High
5	1000 INR	Government hospitals	Support	No vaccinated peers	2	Yes—Mild	Low
6	Free	Government hospitals	Support	Vaccinated peers	1	Yes—Mild	High
7	1000 INR	Private hospitals	Support	Vaccinated peers	2	None	High
8	Free	Private hospitals	Support	No vaccinated peers	1	None	Low

Each scenario was printed on an individual card with associated images to represent the attributes in that scenario. Scenario cards were pilot tested in the community to ensure they were culturally relevant and identifiable.

**Fig. 1:**
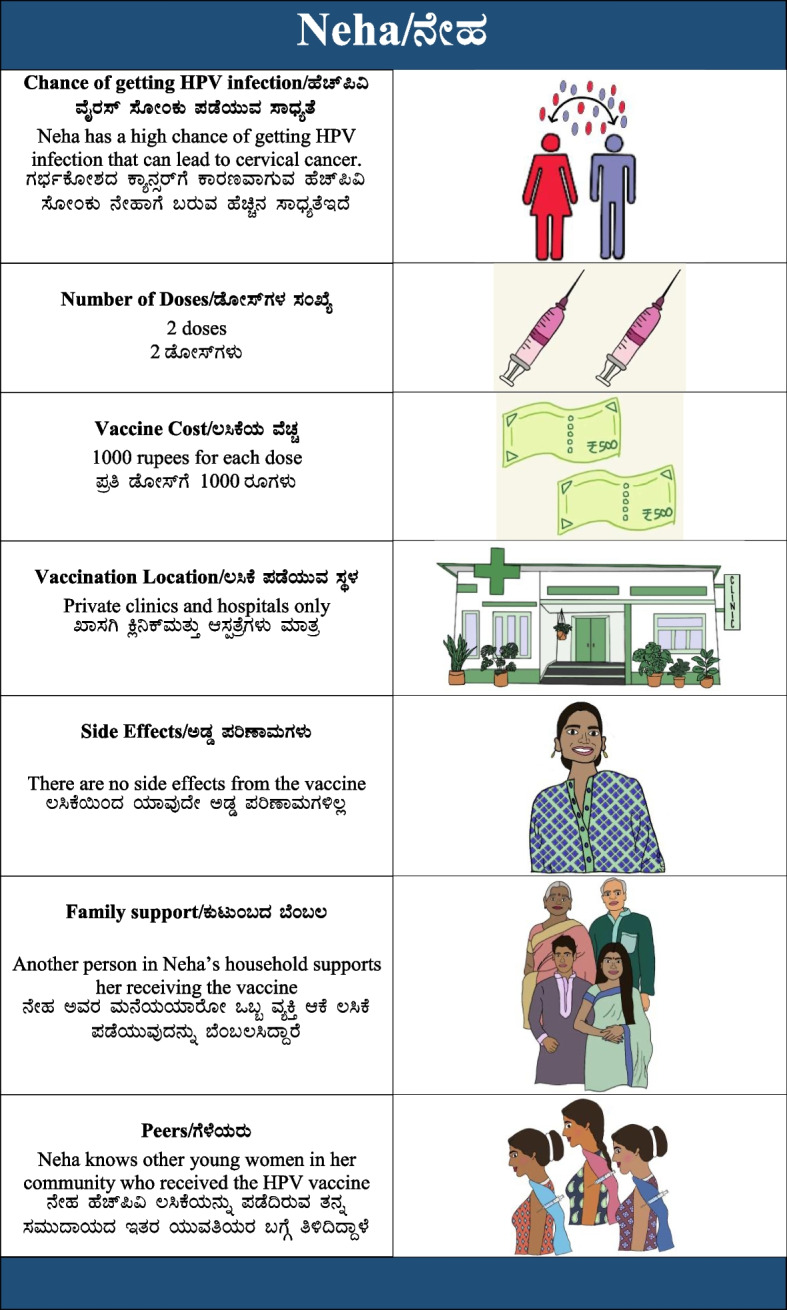
Sample HPV vaccination scenario card from conjoint analysis

Conjoint analysis was administered by trained PHRII research assistants in private settings in the community. Conjoint analysis participants underwent informed consent process, provided basic demographic information, and answered survey questions on prior knowledge of cervical cancer and HPV. They then received the same educational session on cervical cancer, HPV, and HPV vaccination as FGD participants. Following this, participants were presented with the eight hypothetical HPV vaccination scenario cards. Each card was labeled with the name of a “character” to represent a young woman (i.e., Latha, Mamta, Parimala, etc.) considering taking the HPV vaccine (see Fig. 1). Participants were asked to rate how likely each character would be to accept the HPV vaccine in her respective scenario by placing the scenario cards on a 5-point Likert scale, from “Highly unlikely” to “Highly likely” (i.e., “How likely would Latha be to take the HPV vaccine?”). Emoticons were used to represent each of the choices on the Likert scale in order to improve readability and comprehensibility. Because no single scenario contained all the preferred or all the non-preferred attributes, conjoint analysis required participants to weigh and prioritize which attributes were most important to them (for instance, a participant who prioritized cost might rate the scenarios in which the vaccine was free as “Highly likely” to vaccinate, even if other attributes were less favorable). Considering multiple attributes at once also more closely mimics real-world decision-making than asking about each attribute individually.

For the eight HPV vaccination scenarios, ratings from 101 participants were averaged to obtain mean LTV for each scenario. Sample size for conjoint analysis was selected based on literature review and is consistent with other similar conjoint analysis pilots that have generated statistically meaningful results [43–46]. Impact scores were then calculated in two steps to determine the effect of individual attributes on overall LTV. In step 1, a multiple regression model was fit to each respondent’s LTV for HPV vaccination scores *Y*_*i*_ for the eight hypothetical scenarios, *i* = 1….8; the seven HPV vaccination attributes *A*_*p*_, *p* = 1….7 were designated as preferred (1) or not preferred (0) and were independent variables:

$${Y}_{i}={\beta }_{0}+\sum {\beta }_{p}{A}_{p}+{\varepsilon }_{i}$$

In this model, *ɛ*_*i*_ was a residual error term and *β*_*p*_ represented the seven regression coefficients for each HPV vaccination attribute. The regression coefficient equaled the impact score for a given attribute on LTV for each individual participant. The regression model was not adjusted for additional variables. In step 2, individual impact scores for a given attribute were averaged across all respondents, which gave the overall impact of that attribute on LTV against HPV. A one-sample t-test was used to calculate whether each HPV vaccination attribute had a statistically significant impact on LTV.

## Results

Fifty-two participants (*n* = 31 women, *n* = 21 men) took part in seven gender stratified FGDs between September to November 2022. Conjoint analysis survey was administered to 101 women in April 2023. Mean age of all participants was 22.5 years, 66.7% had ever been married, 49.7% had at least one child, and 100% were Hindu. All participants had passed high school (9th grade) and 76.9% were middle- or lower-class based on the BG Prasad socioeconomic scale (Table 2) [47].

**Table 2:** Sociodemographic characteristics of study participants (*n* = 153)

	**FGD men (*n* = 21)**	**FGD women (*n* = 31)**	**Conjoint analysis women (*n* = 101)**
**Age in years, mean (SD)**	22.6 (± 2.4)	23.3 (± 2.5)	22.2 (± 2.8)
**Ever married, n (%)**	12 (57%)	22 (71%)	68 (67%)
**Children, n (%)**	2 (10%)	20 (65%)	54 (53%)
**Religion—Hindu, n (%)**	21 (100%)	31 (100%)	101 (100%)
**Caste, n (%)**			
General Caste	8 (38%)	14 (45%)	63 (62%)
ST/SC/OBC/other^1^	13 (62%)	17 (55%)	38 (38%)
**Highest level of education, n (%)**			
High school or secondary school	13 (62%)	20 (65%)	78 (77%)
Graduate, post graduate, or professional degree	8 (38%)	11 (35%)	23 (23%)
**Socioeconomic class, n (%)**^**2**^			
Upper- or upper middle-class	8 (44%)	7 (24%)	19 (19%)
Middle-class	4 (22%)	8 (28%)	33 (33%)
Lower- or lower middle-class	6 (33%)	14 (48%)	48 (48%)
**Employment status, n (%)**			
Employed full- or part-time	14 (67%)	1 (3%)	1 (1%)
Student	2 (10%)	4 (13%)	27 (27%)
Homemaker	0 (0%)	21 (68%)	63 (62%)
Not employed/other	5 (24%)	5 (16%)	10 (10%)
**With whom do you live? n (%)**^**3**^			
Spouse	0 (0%)	4 (13%)	7 (7%)
Spouse's family	7 (41%)	16 (53%)	57 (57%)
Parents	9 (53%)	9 (30%)	35 (35%)
Other relative	1 (6%)	1 (3%)	1 (1%)

^1^ST = scheduled tribe; SC = scheduled caste; OBC = other backward caste

^2^*n* = 18 FGD men, *n* = 29 FGD women, *n* = 100 conjoint analysis women. Calculated using the BG Prasad scale for SES classification

^3^*n* = 17 FGD men, *n* = 30 FGD women, *n* = 100 conjoint analysis women

**Fig. 2:**
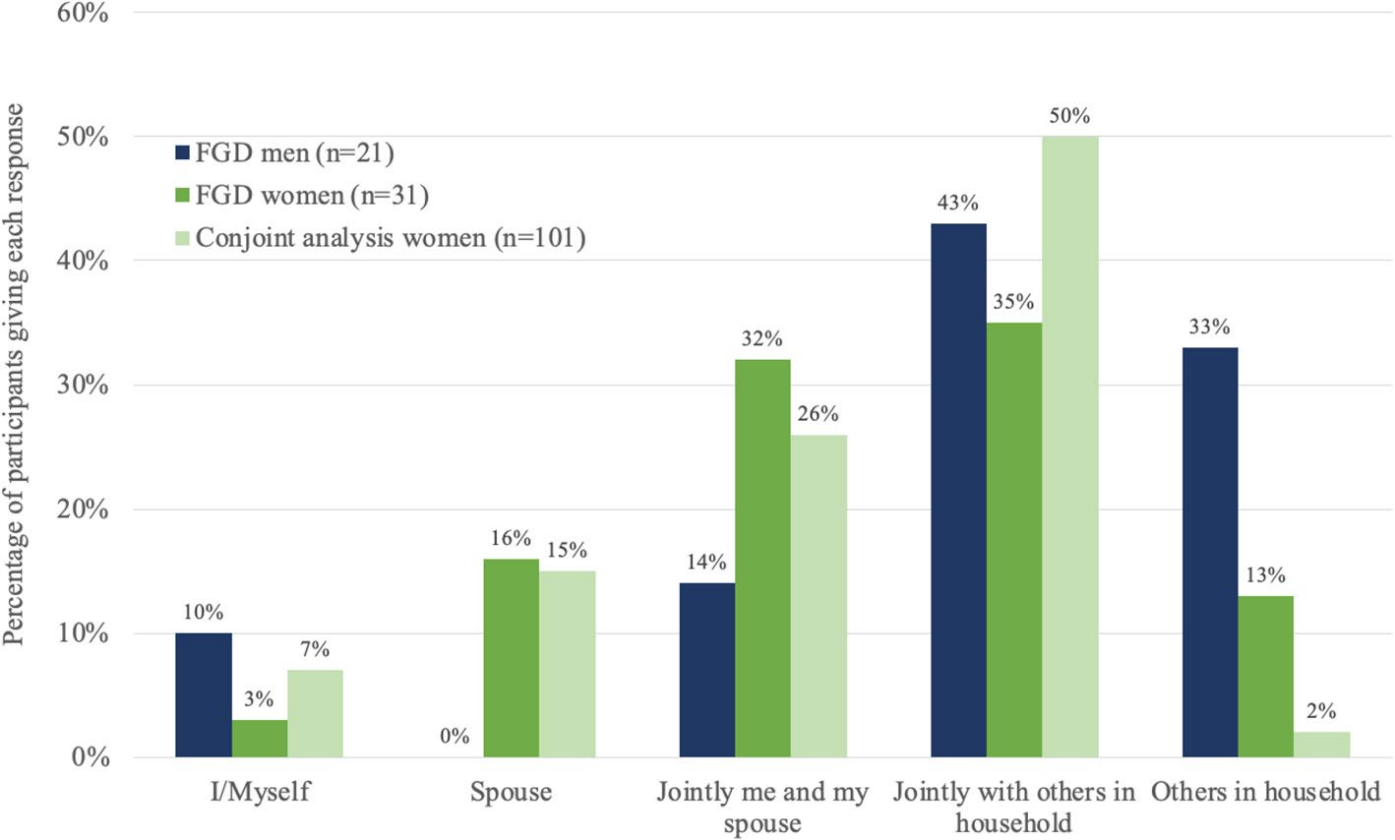
Healthcare decision-making: Who makes decisions about your healthcare?

Out of all participants surveyed, 17.9% had heard of cervical cancer prior to enrolling in the study, and 0.7% knew that HPV causes cervical cancer. All participants had “no information” or “a little information” about HPV. Only 2.7% had heard of the HPV vaccine, and no participants were aware of anybody in their community who had received the vaccine. Amongst young women, 93.9% reported decisions about their healthcare were made either entirely by another member of their household or made jointly with another household member (Table 3, Fig. 2).

**Table 3:** Pre-study knowledge of cervical cancer, HPV, and the HPV vaccine (*n* = 153)

	**FGD men (*n* = 21)**	**FGD women (*n* = 31)**	**Conjoint analysis women (*n* = 101)**
**Had you heard of cervical cancer? n (%)**^**1**^			
Yes	4 (20%)	10 (33%)	13 (13%)
No/Don't know	16 (80%)	20 (67%)	88 (87%)
**Were you aware that HPV causes cervical cancer?**			
Yes	0 (0%)	1 (3%)	0 (0%)
No/Don't know	21 (100%)	30 (97%)	101 (100%)
**How much information did you have about HPV? n (%)**			
No information	20 (95%)	30 (97%)	94 (93%)
A little information	1 (5%)	1 (3%)	7 (7%)
Some or a lot of information	0 (0%)	0 (0%)	0 (0%)
**Were you aware that HPV is transmitted through sexual activity? n (%)**			
Yes	0 (0%)	0 (0%)	2 (2%)
No/Don't know	21 (100%)	31 (100%)	99 (98%)
**Had you heard of the HPV vaccine? n (%)**^**2**^			
Yes	2 (11%)	1 (3%)	1 (1%)
No/Don't know	17 (89%)	30 (97%)	99 (99%)
**How much information did you have about the HPV vaccine? n (%)**			
No information	19 (90%)	29 (94%)	99 (98%)
A little information	2 (10%)	2 (6%)	2 (2%)
Some or a lot of information	0 (0%)	0 (0%)	0 (0%)
**Do you know anybody who has received the HPV vaccine? n (%)**^**3**^			
Yes	0 (0%)	0 (0%)	0 (0%)
No/Don't know	21 (100%)	31 (100%)	99 (100%)
**Were you aware that HPV causes other types of cancer (head and neck, vaginal, vulvar, penile, anal)? n(%)**^**4**^			
Yes	1 (5%)	0 (0%)	1 (1%)
No/Don't know	20 (95%)	30 (100%)	99 (99%)

^1^*n* = 20 FGD men, *n* = 30 FGD women

^2^*n* = 19 FGD men, *n* = 100 conjoint analysis women

^3^*n* = 99 conjoint analysis women

^4^*n* = 30 FGD women, *n* = 100 conjoint analysis women

### Focus Group Discussions

**Fig. 3:**
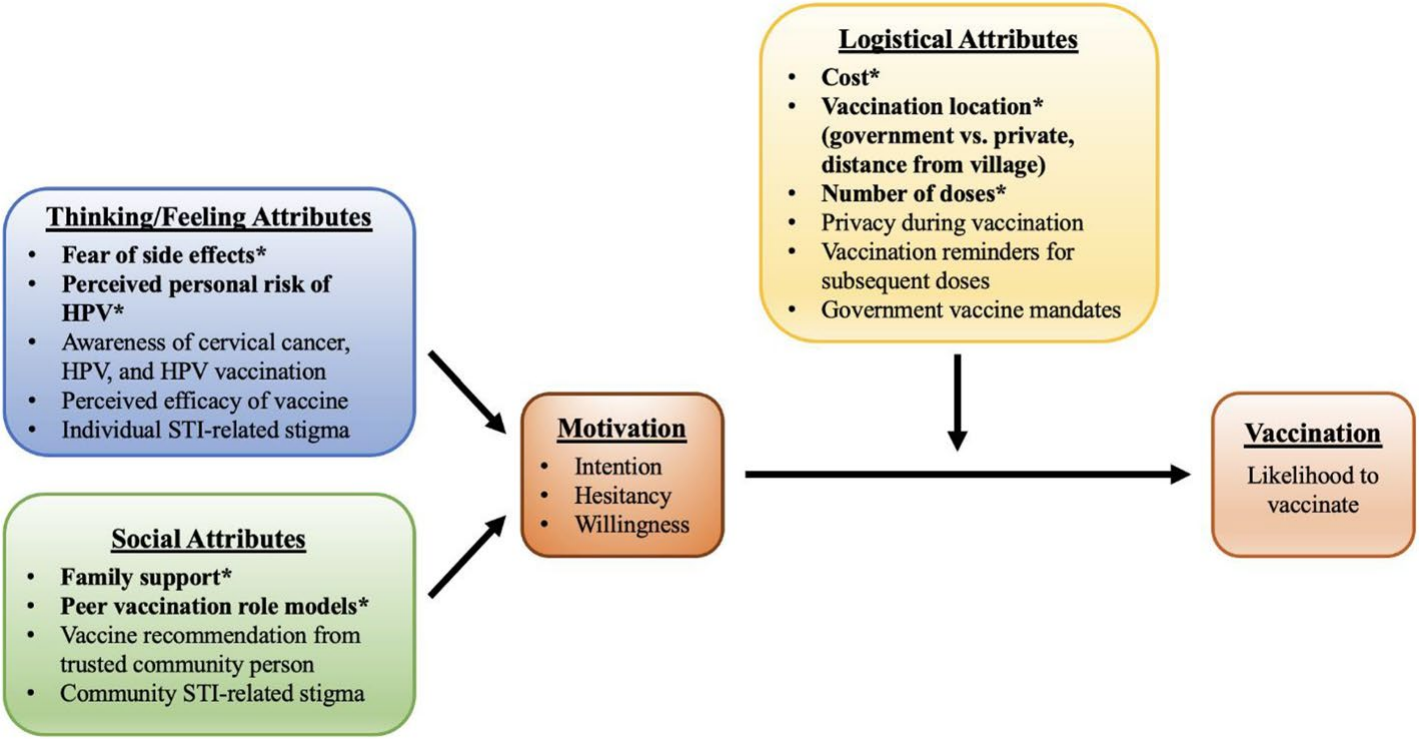
Model for HPV vaccine acceptability amongst young women in rural Mysore, India^a,b^ ^a^Adapted from the Increasing Vaccination Model by Brewer et al. and the WHO behavioural and social drivers of vaccination framework ^b^Figure generated from thematic analysis of qualitative data from focus group discussions *Attributes explored through conjoint analysis

HPV vaccination attributes that emerged during analysis of FGDs are presented in Fig. 3. Attributes in our adapted model were categorized as thinking/feeling attributes, social attributes, or logistical attributes, which correspond to Brewer’s categories of what people think and feel, social processes, and direct behavior changes, respectively; [35, 36] as with the BeSD framework, we include motivation as a separate domain. Qualitative data for select attributes are summarized below.

### Thinking/Feeling Attributes

#### Fear of side effects

While participants felt that general vaccine-related side effects such as swelling, nausea, and joint pain could deter some people from vaccinating, they were more concerned about the possibility of gynecologic side effects given HPV infects the cervix. Potential infertility from vaccination was a commonly cited concern for both men and women.

*“We have fear that we may not have children in future. If we get vaccinated it might damage the uterus.” – FGD 4 (women)*

#### Perceived personal risk of HPV

Women with multiple sexual partners and early marriage were perceived to be at high risk for HPV. Most women who participated in FGDs did not perceive themselves personally to be at risk; this was particularly true for unmarried women, and participants suggested that family and community members may not understand the need to vaccinate unmarried women against HPV.

*“They might say that you have not been married, nor have had [sexual] interaction, why should you take the vaccine?” – FGD 3 (women)*

#### Awareness of cervical cancer, HPV, and HPV vaccination

Men and women suggested several strategies to mitigate low awareness of cervical cancer, HPV, and HPV vaccination in their communities. In addition to in-person education from community health workers, participants requested dissemination of information through mobile phones and social media, radio/TV, street plays, camps, village “mikings” (village announcements over megaphones), and schools as part of the reproductive health curriculum (along with HIV). Inclusion of videos and visual aids were strongly preferred, as were use of stories to illustrate the impact of cervical cancer on community people’s lives.

*“Nowadays we can reach them through the media. Everyone has a mobile [cell phone], women are on the mobile a lot, because of that we can reach them faster.” – FGD 1 (women)*

*“In school and colleges, we have to pass the information through children so that they go home and discuss the same.” – FGD 7 (men)*

### Social Attributes

#### Family support

Support from husbands, in-laws, parents, and elder household members was considered essential for vaccination, especially because young women might need family assistance if the HPV vaccine caused side effects.

*“If there are any side effects after taking the injection [vaccine], the one who looks after us is our family. That is why there should be support.” – FGD 2 (women)*

*“In joint family, if one member says no, then everyone will say no.” – FGD 5 (men)*

#### Peer influence

Participants felt that seeing other young women from their community take the HPV vaccine and receiving information about the vaccine from peers would be an important facilitator for vaccination.

*“They [people] are listening to what others are saying. If I come to know that you have gotten it, only then will I get it as well. If I get it, she will get it.” – FGD 3 (women)*

#### Recommendations for vaccination

Men and women identified several trusted community persons whose recommendation would be most effective in increasing uptake of the HPV vaccine. These included Accredited Social Health Activists (ASHAs)—local community health workers who are responsible for disseminating health-related information in rural areas; Anganwadi (rural childcare center) teachers; and local doctors from government centers. Men additionally expressed trust in the recommendations of village chiefs.

*“Village people will believe ASHA workers more…Since the village people themselves become ASHA workers, they will trust them.” – FGD 3 (women)*

#### Community STI-related stigma

Both men and women believed that community gossip could deter women from taking the HPV vaccine, especially because some community members might assume that women who receive the HPV vaccine have extramarital sexual partners.

*““She might have other sexual contact that is why she got vaccinated,” this is how the society may think.” – FGD 5 (men)*

*“They will say that she has got [HPV] because she has sexual interaction with everyone…Since they don’t know the exact reason, they will create a false narrative.” – FGD 3 (women)*

### Logistical Attributes

#### Cost

Given financial hardships faced by many families in rural areas as well as the precedent set by free COVID-19 vaccination, participants expressed doubt that families would pay for young women to receive the HPV vaccine.

*“Village people will not agree if they will have to pay for vaccine…in my home, we do not have money and that is a problem. We will think that we cannot afford it today, so we will take it some other time.” – FGD 6 (men)*

#### Vaccination location

Government hospitals and subcenters were the preferred location to receive the HPV vaccine, rather than private clinics. Government subcenters were described as easily accessible for rural people. Participants additionally perceived vaccines administered at government centers to be government-certified and thus safe to take; all agreed that including the HPV vaccine in the National Immunization Program for young women would legitimize the vaccine and encourage uptake.

*“If it is in the government hospital, it will be certified. So, we can get the vaccine over there.” – FGD 2 (women)*

### Conjoint analysis

Conjoint analysis results were calculated for *n* = 101 participants who rated the eight hypothetical vaccination scenarios. Mean LTV on a 100-point scale for each scenario are shown in Table 4. Among the eight scenarios, LTV ranged from 42.57 to 97.03, with higher numbers indicating greater likelihood of vaccination. The scenario with the highest LTV had the following attribute profile: “*Mamta is at high risk for acquiring HPV. She will receive one dose of the vaccine, free of cost, at a government hospital/subcenter. She will experience minor side effects (fever, nausea, joint pain). Mamta’s family supports her receiving the vaccine, and she knows other young women in her community who received the vaccine*.” The scenario with the lowest LTV had the following attribute profile: “*Jaya is at low risk for acquiring HPV. She will receive one dose of the vaccine at a cost of 1,000 rupees at a private hospital. She will experience minor side effects (fever, nausea, joint pain). Nobody in Jaya’s family supports her receiving the vaccine, but she knows other young women in her community who received the vaccine*.”

**Table 4:** Likelihood to vaccinate (LTV) against HPV for eight hypothetical vaccination scenarios in Mysore, India (*n* = 101)

	*Attributes*						
**Mean LTV (SD)**	**Cost**	**Location**	**Family support**	**Peer influence**	**Dose number**	**Side effects**	**HPV risk**
97.03 (8.1)	Free	Government hospitals	Support	Vaccinated peers	1	Yes—Mild	High
81.68 (25.2)	1000 INR	Private hospitals	Support	Vaccinated peers	2	None	High
72.77 (29.2)	Free	Government hospitals	None	Vaccinated peers	2	None	Low
68.56 (30.5)	Free	Private hospitals	Support	No vaccinated peers	1	None	Low
52.97 (34.9)	Free	Private hospitals	None	No vaccinated peers	2	Yes—Mild	High
50.99 (35.5)	1000 INR	Government hospitals	None	No vaccinated peers	1	None	High
49.50 (32.4)	1000 INR	Government hospitals	Support	No vaccinated peers	2	Yes—Mild	Low
42.57 (31.3)	1000 INR	Private hospitals	None	Vaccinated peers	1	Yes—Mild	Low

The impact of each HPV vaccination attribute on LTV is shown in Table 5. For the overall cohort, dose number was the only attribute that did not have a statistically significant impact on LTV. Family support (impact score = 19.37, *p* < 0.0001) and peer influence (impact score = 18.01, *p* < 0.0001) had the greatest overall impact on LTV, followed by cost (impact score = 16.64, *p* < 0.0001) and personal risk of HPV (impact score = 12.31, *p* < 0.0001). When stratified by age and marital status, family support and peer influence had a relatively greater impact on LTV for never-married women (impact scores = 26.89, *p* < 0.0001 (family support) and 22.73, *p* < 0.0001 (peer influence)) and younger women (impact scores = 21.22, *p* < 0.0001 (family support) and 20.96, *p* < 0.0001 (peer influence)). Side effects were more impactful among never-married women (impact score = 16.7, *p* < 0.0001), moving ahead of HPV risk. By contrast, side effects were not a statistically significant predictor of LTV for ever-married women. HPV risk had a greater impact among older women (impact score = 16.04, *p* < 0.0001), moving into second place behind family support, and among ever-married women (impact score = 14.61, *p* < 0.0001) compared to younger and never-married women.

**Table 5:** Impact of HPV vaccination attributes on likelihood to vaccinate (LTV) for young women in Mysore, India

***Total (n = 101)***			
HPV vaccination attributes	Preferred value mean LTV^a^	Non-preferred value mean LTV^b^	Impact on LTV mean (SD)^c^
Family support	74.2	54.83	19.37 (23.8)*
Peer influence	73.51	55.51	18.01 (19.6)*
Cost	72.83	56.19	16.64 (19.3)*
HPV risk	70.67	58.35	12.31 (23.1)*
Side effects	68.5	60.52	7.98 (20.9)*
Location	67.57	61.45	6.13 (18.4)*
Dose number	64.79	64.23	0.56 (18.6)
***Stratified by Marital Status***			
*Never married (n* = *33)*		*Ever married (n* = *68)*	
HPV vaccination attributes	Impact on LTV mean (SD)^c^	HPV vaccination attributes	Impact on LTV mean (SD)^c^
Family support	26.89 (18.5)*	Family support	15.72 (25.4)*
Peer influence	22.73 (16.2)*	Peer influence	15.72 (20.8)*
Cost	21.59 (18.7)*	HPV Risk	14.61 (25.4)*
Side effects	16.7 (18.9)*	Cost	14.25 (19.3)*
HPV Risk	7.58 (16.5)*	Location	5.42 (19.2)*
Location	7.58 (16.7)*	Side effects	3.77 (20.7)
Dose number	-4.17 (17.0)	Dose number	2.85 (19.0)
***Stratified by Age***			
*Age 18–22 (n* = *48)*		*Age 23–26 (n* = *53)*	
HPV vaccination attributes	Impact on LTV mean (SD)^c^	HPV vaccination attributes	Impact on LTV mean (SD)^c^
Family support	21.22 (25.0)*	Family support	17.69 (22.9)*
Peer influence	20.96 (20.6)*	HPV risk	16.04 (24.6)*
Cost	18.36 (19.2)*	Peer influence	15.33 (18.4)*
HPV risk	8.20 (20.8)*	Cost	15.09 (19.5)*
Side effects	7.42 (22.9)*	Side effects	8.49 (19.1)*
Location	5.34 (18.9)	Location	6.84 (18.0)*
Dose number	-0.13 (19.9)	Dose number	1.18 (17.5)

^a^Average LTV score for the four scenarios with the preferred attribute value

^b^Average LTV score for the four scenarios with the non-preferred attribute value

^c^Impact on LTV is calculated by taking the difference between the mean LTV for scenarios with the preferred attribute value and the mean LTV for scenarios with the non-preferred attribute value

^*^p < 0.05 for the impact of the vaccination attribute on mean LTV score, calculated using one-sample t-test

## Discussion

Our mixed-methods study in rural Mysore, India revealed low to non-existent knowledge of cervical cancer, HPV, and the HPV vaccine amongst young men and women. Participants identified a critical need for education in their villages. After providing basic awareness about the HPV vaccine to participants, multiple attributes were found to influence young women’s willingness to accept vaccination. Social attributes, including support from key family members and knowing peers who had taken the vaccine, had the greatest impact upon LTV, especially among younger women and women who have never married. Logistical attributes (vaccine cost, location of vaccination) and thinking/feeling attributes (perceived personal risk of HPV, side effects) were also significant vaccination predictors. Participants expressed strong preferences for involvement of trusted community stakeholders in the implementation of future vaccination programs.

Although our study cohort was well-educated, with 97.7% of young women having finished 12th grade education, pre-existing knowledge of cervical cancer, HPV as a cause of cervical cancer, and HPV vaccination amongst female participants were 17.6%, 0.8%, and 1.5%, respectively. These levels are generally lower than other surveys conducted amongst women in India, [22, 23, 48, 49] which may reflect the rural location of our study [23, 25]. Knowledge was also low amongst young men in our study. In India, men’s knowledge of women’s health issues is particularly salient because many women live in traditional multi-generational joint family households, especially in rural areas [34]. Decisions about women’s healthcare and vaccination are thus often made at the household level, with input from husbands, in-laws, and other family members [50–52]. In our cohort, the vast majority of women indicated involvement of other family members in healthcare decision-making.

Conjoint analysis further highlighted the critical importance of engaging women’s social circles to encourage HPV uptake, as family support and peer influence were the two attributes with the greatest impact on LTV. Given low levels of knowledge for both men and women, targeted education campaigns are needed to increase awareness of cervical cancer and HPV for young women and their families. A recent study in rural India piloted a family-centered sexual health education intervention to improve uptake of HPV testing for women, providing story-based audio-visual education to both women and a male family member; the intervention demonstrated improved attitudes and knowledge regarding cervical cancer screening and reduced STI-related stigma [53]. Similar family-based approaches should be applied to HPV vaccination. Presentation of information in multiple mediums may improve engagement and increase accessibility for low-literacy groups; [54, 55] in another study of online administration of HPV educational materials to Indian parents, short videos were found to be the most effective means of increasing awareness [56]. The expanded use of mobile Internet technology to access health information in rural India offers a potential avenue to deliver education to community members [57]. As interventions are implemented, peer influence could be harnessed by enlisting vaccinated young women as peer educators to provide information and encourage uptake within their communities [54, 58]. Overall, existing literature on non-biomedical, social support-based interventions for increasing HPV vaccine uptake is sparse, and this represents a critical area of future investigation.

Two thinking/feeling attributes from our model significantly impacted LTV: personal risk of HPV (especially amongst older and ever-married women) and side effects (especially never-married women). The disparity of HPV risk impact scores between these two groups likely reflects that older, married women perceive their own risk of HPV to be higher. Our qualitative data suggest that community members may not understand the need to vaccinate low-risk, unmarried women, which is consistent with other literature from India [59, 60]. Minor side effects was a significant negative predictor of LTV for unmarried women in particular, and FGD participants expressed concern about more serious gynecologic and fertility-related side effects, despite receiving education about the well-established safety record of HPV vaccines [61]. Finally, although not explored in our conjoint analysis, participants in FGDs believed stigma around STIs could limit uptake [7]. Family-centered interventions should be specifically tailored to mitigate common fears (i.e. risk of fertility-related side effects) and misconceptions (i.e. lack of need to vaccinate unmarried women), and should incorporate anti-stigma messaging.

Of the logistical attributes presented in conjoint analysis, cost had the greatest impact upon LTV, followed by vaccination location, with participants favoring vaccination at government centers as part of the National Immunization Program. Number of doses had no significant impact. Most participants were middle class or below in the socioeconomic strata, meaning their per-capita monthly income was less than 4,329 INR (53 USD); [47] this is less than the average per-capita income of Mysore District and may reflect lower income-earning opportunities in rural areas [62]. At its current estimated cost of 1,000 INR per dose, the Serum Institute of India’s new vaccine likely remains unaffordable for many rural families. Cost is perceived by healthcare providers in India to be a major barrier to HPV vaccine uptake, [63] and having an HPV vaccine “freely available from the government sector” would positively impact physicians’ decision to recommend vaccination [64]. Adult vaccine hesitancy in India has been associated with poor national guidelines for adult immunizations [65]. Providing formal catch-up vaccination recommendations as part of the National Immunization Program, with the vaccine available free or partially-subsidized at government centers, would increase acceptability of HPV catch-up vaccination.

When discussing dissemination of vaccine-related information and implementation of recommendations, FGD participants expressed strong confidence in local stakeholders such as ASHAs, Anganwadi teachers, doctors, and village chiefs (particularly for men). As trusted members of their local communities, ASHAs and Anganwadi teachers are instrumental in providing frontline health services in rural India, [66–68] and both played important roles in combating the COVID-19 pandemic, including coordinating vaccination efforts [68, 69]. The infrastructure and networks established to accelerate administration of the COVID-19 vaccine in rural India should be utilized in HPV vaccine roll-out [70]. Including local stakeholders in devising HPV vaccination interventions will be critical for program success in Indian communities [71]. In the long-term, adding HPV and cervical cancer as subjects in standard science curriculum should also be considered.

This study has several limitations. First, the small sample size, restriction of sampling to rural areas, religious homogeneity, and high educational attainment of our cohort may limit the generalizability of these findings to other groups of young women in South India. Further research is needed on how religion may affect HPV vaccine uptake, vaccine acceptability in groups with low literacy, and knowledge and acceptability of HPV vaccination amongst young women living in urban areas. Secondly, because most participants had no knowledge of cervical cancer or HPV, education was provided to participants immediately prior to administering the conjoint analysis survey. Durability of knowledge, attitudes toward vaccination, and vaccination intentions should be assessed in future studies. Thirdly, this is an observational study and no causal inference can be drawn from this study.

This study also has several strengths. It is the first study in India to employ conjoint analysis to better understand contributors to HPV vaccination decision-making. Navigating the best approaches for HPV vaccination and discussing the illnesses it prevents are particularly complex given the cultural and social norms that influence women’s health in India. We believe that by using a mixed-methods approach, we were able to gauge community-level perceptions about the HPV vaccine while objectively measuring individual-level factors that affect a woman’s decision to take it. We were able to produce robust findings that can be translated into policy or incorporated into existing strategies with the upcoming roll-out of India’s first indigenous HPV vaccine.

## Conclusion

Participants from rural Mysore had poor knowledge of cervical cancer, HPV, and HPV vaccination. Social attributes had the greatest impact on young women’s LTV, and future interventions should prioritize family-based approaches and peer education to increase HPV vaccine uptake, with awareness campaigns tailored to address safety concerns. Cost was the most impactful non-social attribute, highlighting the need for free or subsidized vaccination. Mobilizing local stakeholders, offering vaccination at government centers, and incorporating official recommendations for catch-up HPV vaccination into the National Immunization Program would maximize uptake amongst rural young women.

## Data Availability

The datasets used and/or analysed during the current study are available from the corresponding author on reasonable request.

## Abbreviations

HPV: Human papillomavirus
WHO: World Health Organization
FGD: Focus group discussion
LTV: Likelihood to vaccinate
STI: Sexually transmitted infection
PHRII: Public Health Research Institute of India
IVM: Increasing vaccination model
BeSD framework: WHO behavioural and social drivers of vaccination framework
INR: Indian rupees
USD: U.S. dollars
ASHA: Accredited Social Health Activists
